# The Role of Metal Binding in the Amyotrophic Lateral Sclerosis-Related Aggregation of Copper-Zinc Superoxide Dismutase

**DOI:** 10.3390/molecules22091429

**Published:** 2017-08-29

**Authors:** Ivana Sirangelo, Clara Iannuzzi

**Affiliations:** Department of Biochemistry, Biophysics and General Pathology, Università degli Studi della Campania “Luigi Vanvitelli”, 80138 Naples, Italy; ivana.sirangelo@unicampania.it

**Keywords:** metals, protein misfolding, SOD1, neurodegeneration, amyloid aggregation

## Abstract

Protein misfolding and conformational changes are common hallmarks in many neurodegenerative diseases involving formation and deposition of toxic protein aggregates. Although many players are involved in the in vivo protein aggregation, physiological factors such as labile metal ions within the cellular environment are likely to play a key role. In this review, we elucidate the role of metal binding in the aggregation process of copper-zinc superoxide dismutase (SOD1) associated to amyotrophic lateral sclerosis (ALS). SOD1 is an extremely stable Cu-Zn metalloprotein in which metal binding is crucial for folding, enzymatic activity and maintenance of the native conformation. Indeed, demetalation in SOD1 is known to induce misfolding and aggregation in physiological conditions in vitro suggesting that metal binding could play a key role in the pathological aggregation of SOD1. In addition, this study includes recent advances on the role of aberrant metal coordination in promoting SOD1 aggregation, highlighting the influence of metal ion homeostasis in pathologic aggregation processes.

## 1. Introduction

**Figure 1:**
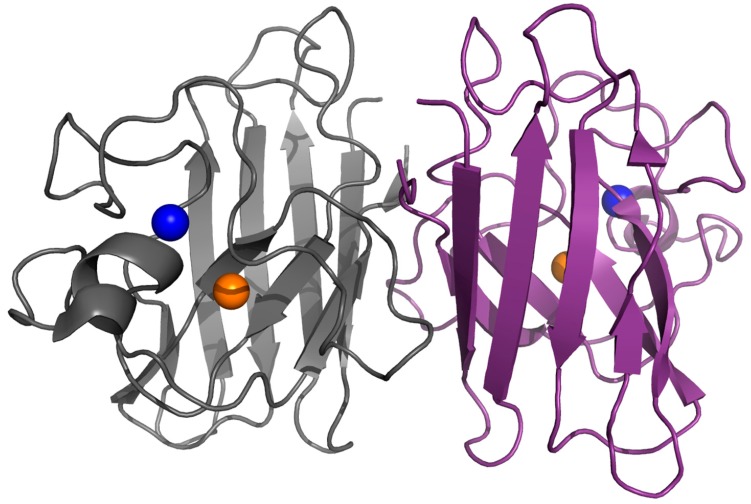
Dimeric SOD1. Structure of human dimeric copper-zinc superoxide dismutase (PDB code: 1l3n). The copper and zinc ions are represented in orange and blue respectively.

Protein misfolding, aggregation, and formation of insoluble amyloid deposits are common pathological hallmarks in many neurological disorders such as Huntington’s, Alzheimer’s, and Parkinson’s diseases and amyotrophic lateral sclerosis (ALS) [1]. In these conformational diseases, misfolding and aggregation of proteins seem to be directly related to neurotoxicity [2,3]. In amyloid diseases, a normally soluble protein forms insoluble fibrillary structures that share common structural features despite the considerable diversity in the primary sequence of the component proteins. Indeed, amyloid fibrils are typically composed of unbranched fibrils rich in β-sheet structures in which the ordered regions adopt a cross-β structure [4,5]. Although the aggregation process of proteins has been widely studied in vitro and many physiological factors have been identified, the molecular determinants that promote or modulate the abnormal protein-protein interactions, leading to protein aggregation, remain elusive. Among them, metal ions seem to play a key role in amyloid aggregation as they were shown to be directly involved in many neurodegenerative disorders like Alzheimer’s, Parkinson’s and prion diseases. In particular, metal binding has been shown to strongly affect both protein oligomerization and neurotoxicity in β-amyloid peptide, α-synuclein, prion protein, human islet amyloid polypeptide and superoxide dismutase 1 [6,7,8,9,10,11]. In addition, metal ion homeostasis is a hallmark in neurodegenerative conditions [12]. Eukaryotic copper-zinc superoxide dismutase 1 (SOD1) is a 32-kDa homodimeric metalloenzyme and each homodimer contains an active site that binds a catalytic copper ion and a structural zinc ion (Figure 1). The mature, correctly folded and enzymatically active form is obtained in vivo through several post-translational modifications: binding of zinc and copper ions, disulfide bond formation, and dimerization [13,14,15]. SOD1 is implicated in the formation of proteinaceous toxic aggregates in the affected motor neurons of familial and sporadic forms of ALS [16,17,18,19,20,21,22,23]. Indeed, SOD1 is an extremely stable protein when fully assembled, but it is rather unstable and prone to aggregation in its metal-free form. Many studies have shown that wild-type human SOD1, when lacking both its metal ions, forms large and stable amyloid-like protein aggregates under physiological conditions of pH and temperature thus suggesting that metal binding could play a key role in the in vivo aggregation process of SOD1 [15,24,25,26,27,28,29]. Moreover, it has been reported that also some SOD1 mutants, many of them related to familial ALS, form amyloid aggregates both in vitro and in vivo and that demetalation is the key factor for aggregation [15,25,29,30,31,32,33,34,35,36].

This review discusses the role of metal binding in the aggregation process of SOD1 in familial and sporadic ALS. In addition, we report recent advances on the role of aberrant metal coordination in promoting SOD1 aggregation, highlighting the influence of metal ion homeostasis in pathologic aggregation processes.

## 2. Structure, Folding and Stability in SOD1

**Figure 2:**
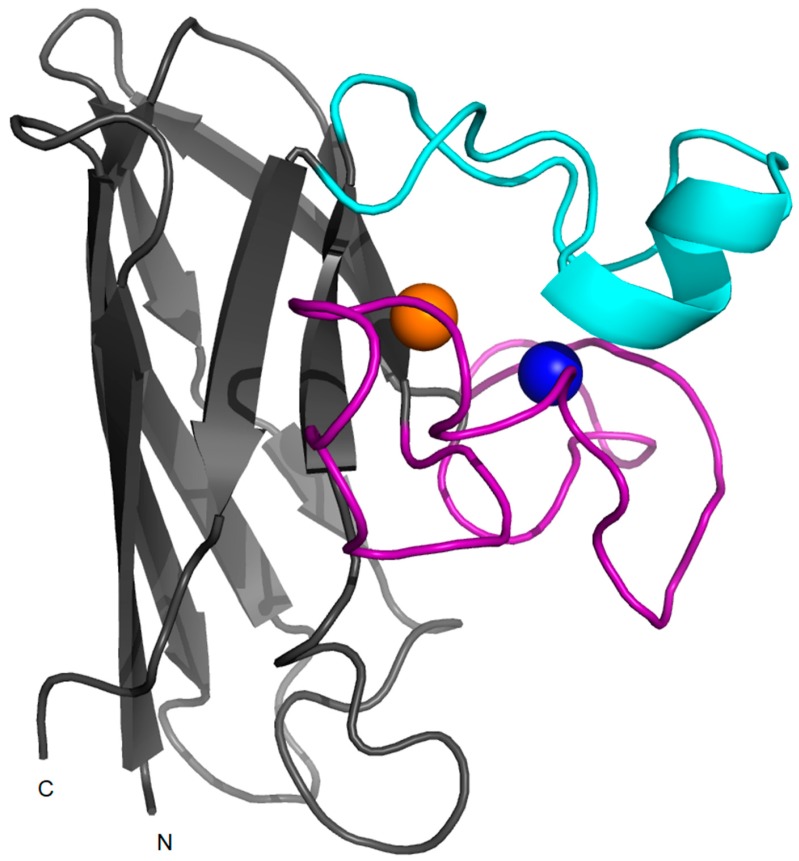
Monomeric SOD1. Structure of a monomer of human wild-type SOD1 (PDB code: 1l3n) in which copper and zinc ions are represented in orange and blue respectively. The electrostatic loop is shown in cyan and the zinc-loop is shown in magenta.

SOD1 is a ubiquitous protein that functions as a primary antioxidant by catalyzing the disproportionation of superoxide radicals to hydrogen peroxide and molecular oxygen. The homodimeric protein is localized predominantly in the cytoplasm, but it is also found in other cellular compartments including the nucleus, endoplasmic reticulum and mitochondria [37,38]. The human enzyme is a 32-kDa homodimer and each subunit binds one Cu^2+^ and Zn^2+^ in a binuclear site (Figure 1). SOD1 is a 153-residue polypeptide folded into an eight-stranded beta-barrel motif flanked by two major loops, the “electrostatic” and “zinc” loops that together shape the active site pocket. The beta-barrel fold is a fundamental property of the polypeptide and it can be observed even in the absence of metals and disulfide bonds. Zn^2+^ is coordinated to three histidine and an aspartic residue in the zinc loop which is linked to the β-barrel by an intra-subunit disulfide bridge (Figure 2).

Zinc binding plays roles in both maintaining the catalytic activity over a wide pH range and in stabilizing the structure of SOD1 [13,39]. One of the zinc-coordinated histidines is simultaneously coordinated to the copper (in the cupric ion state) by an imidazole side chain and, together with other three histidine residues, keeps the copper in a distorted square planar geometry. The copper ion is responsible for the catalytic activity as, switching between the cupric and cuprous states, it generates hydrogen peroxide and molecular dioxygen from superoxide dismutation. Interestingly, while all the residues that coordinate zinc belong to a flexible loop region, three of the four copper ligands lay in β-strands that are structured even in the absence of metals [13,39,40,41]. In the absence of coordinated Cu^2+^ and Zn^2+^, the β-barrel and the dimer interface remain intact but the loops result more disordered. While the mechanism of zinc acquisition by SOD1 in vivo is still unclear, copper is inserted by a copper chaperone (CCS) concomitantly with the formation of an intrasubunit disulfide bond [42,43,44,45]. This may not be the only mechanism in humans, since SOD1 in mammalian systems can acquire copper also via a CCS-independent mechanism [46].

A key feature of SOD1 structure is the strong contribution of the post-translational modifications to its stability. Indeed, the maturation of the human SOD1 consists of the following steps: N-terminal acetylation (common to most eukaryotic cytoplasmic proteins), the insertion of copper and zinc ions, the formation of a disulfide bond in each subunit, and dimerization [43,44,47]. These steps are likely to be mutually dependent in vivo as the oligomeric state of SOD1 in vitro is dependent on the metalation state and cysteine residues. In particular, it has been shown that wild-type SOD1 is monomeric only in the disulfide-reduced, metal-free apo-state while disulfide bond formation or the binding of a single metal ion (copper or zinc) in the disulfide-reduced state lead to dimer formation. Likewise, the binding of zinc and/or copper has been shown to stabilize the disulfide bond from reduction [48,49,50]. SOD1 is an unusual protein able to maintain the disulfide bond even in the reducing environment of the cytoplasm and the native intrasubunit disulfide bond is known to contribute to its enzymatic activity [51]. Thus, the metal cofactors, by modulating the status of the disulfide bond, contribute to both the enzymatic activity and structural integrity of SOD1.

The contribution of the different post-translational modifications on the stabilization of SOD1 structure has been studied by complementary biophysical techniques. In particular, a study performed by differential scanning calorimetry revealed for the disulfide-reduced, monomeric apoprotein a *T*_m_ of 42 °C; for the more stable dimeric form a T_m_ of 52 °C; and for the disulfide-reduced dimeric form a *T*_m_ of 58 °C obtained by binding one zinc per dimer [49,51]. The different melting temperatures observed for the two different dimeric SOD1 suggest that the binding of a single zinc to the disulfide-reduced form produce a more stable dimer than that obtained upon disulfide bond formation. Zinc binding has been also shown to increase the stability of the disulfide-intact form of SOD1 [52]. Indeed, this study indicated that the zinc-bound subunit stabilizes the apo subunit as suggested by the increase in its melting temperature from 52 °C to 60 °C. Thus, the presence of zinc in a single subunit results in stronger intersubunit interactions compared to the apo dimer likely through a tighter dimer interface [52]. The ability of zinc to enhance subunit affinity has been also suggested by analytical centrifugation experiments showing that metallated SOD1 containing 2.9 equivalents of zinc and 0.6 equivalents of copper dissociates to monomers at 2.0–3.0 M guanidinium hydrochloride (GdmCl) while the apo SOD1 dissociates over the range of 0.5–1.0 M GdmCl [49].

Recently, the zinc binding to apo SOD1 at different stages of its maturation process has been studied by isothermal titration calorimetry. In this study, the authors have characterized the binding of Zn^2+^ to three models of SOD1: oxidized dimer, oxidized monomer and reduced monomer [53]. Results obtained for the oxidized dimer suggest that Zn^2+^ binds to the two zinc sites via dissimilar mechanisms and affinities, while Zn^2+^ binding to the two copper sites in the homodimer occurs through a parallel mechanism with similar energetics. The binding of the first Zn^2+^ per apoprotein homodimer was reported to have a more profound effect on the protein structure than binding of the second Zn^2+^, although the two zinc sites are structurally identical in the homodimer [52]. The study of Leal and coworkers calculated asymmetrical binding stoichiometries for binding of Zn^2+^ to the two zinc binding site suggesting that the initial binding of one zinc to the apo homodimer produces a one-zinc dimer which can then exchange subunits with a second equivalent one-zinc dimer, resulting in a two-zinc homodimer and one apo homodimer [53]. These results suggest that a higher number of Zn^2+^ participate in the binding to the first high affinity zinc binding site. Differently, isothermogram of Zn^2+^ binding to the oxidized SOD1 monomer indicated low binding affinity and suggested that the zinc binding affinity to the oxidized monomer is distinct from that of binding to dimer in agreement with recent NMR findings [53,54]. Finally, the investigation of Zn^2+^ binding to the reduced SOD1 monomer revealed two distinct binding processes. The zinc binding to this SOD1 conformer is particularly significant, as this process has been suggested to occur in the in vivo folding [55,56]. The authors suggest that the first binding event corresponds to the interaction with the relatively solvent-exposed zinc site in the SOD1 monomer that thus strongly binds Zn^2+^ and greatly impacts on SOD1 overall structure, in contrast with the copper site that is yet disorganized [39,56]. The second binding event observed could be due to misligation of Zn^2+^ to the copper site [53]. Indeed, it was shown that exposure of apo SOD1 immature conformers to labile zinc in vitro at physiological pH, generates a SOD1 conformer where Zn^2+^ is bound to the native zinc site but is also aberrantly bound to the copper site, forming a di-zinc protein [43,57]. In contrast, an in cell NMR study revealed that, within the cytoplasm environment, immature SOD1 selectivity coordinates zinc exclusively to the zinc site [58]. In spite of this accurate insertion of zinc, no metallo-chaperone protein that loads zinc into apo SOD1 has yet been found.

The exceptional stability of metallated SOD1 is demonstrated by the ability of this protein to maintain the enzymatic activity even under highly denaturing conditions, such as 6 M GdmCl or 4% SDS [50,59,60]. This unusual behavior is likely due to the high resistance of holo-SOD1 to unfold completely in the presence of GdmCl, as observed by NMR studies [41]. Indeed, 0.5 M GdmCl was shown to induce only small conformational changes in metallated SOD1 while protein unfolding is achieved only at GdmCl concentrations of 3.5 M or higher. Interestingly, the unfolded species still possess a residual globular structure with a partly formed hydrophobic core that persists even at 8 M GdmCl showing that, even in highly denaturing conditions, metallated SOD1 never unfolds to a fully random coil conformation [41]. Apo SOD1 was shown to be less resistant to GdmCl-induced unfolding as it adopts a similar conformation with residual globular structure at 1 M GdmCl. However, for both apo and holo-SOD1, the unfolding process originates from the loops exposed in the structure and proceeds to the β-strands placed in its core thus explaining why metal binding, disulfide formation and dimerization, providing structure to many loops that connect β-strands, strongly increase the thermal stability of SOD1 [41].

## 3. Role of SOD1 Aggregation in ALS

Amyotrophic lateral sclerosis is a neurological disease characterized by the selective death of motor neurons leading to progressive muscle atrophy, paralysis, and eventual death [61]. Although it is predominantly a sporadic disease, 10% of the ALS cases are described as familial (fALS), a dominantly inherited disease in which patients are heterozygous and express both the mutant and the wild-type form of SOD1. Familial forms of the disease are caused by mutations in a number of proteins, of which SOD1 represents the best-studied example. A link between fALS and mutations in the SOD1 gene was first suggested in 1993 [62], and over 100 fALS-linked mutations, distributed throughout the SOD1 gene, are now associated with approximately 20% of the fALS cases [13,61,63]. These are predominantly single amino acid substitutions although deletions, insertions, and C-terminal truncations also occur. Mutations are distributed along the entire length of the polypeptide and affect the onset, duration, and severity of symptoms in afflicted individuals. The pathogenicity of SOD1 mutants has been shown to be due to the gain of a toxic function and not to the loss of the normal function. Indeed, SOD1 knock-out mice do not show any ALS symptoms, whereas transgenic mice, expressing, for example, the fALS-associated mutant G93A human SOD1, develop the symptoms, despite expression of endogenous mouse SOD1 [64,65,66,67,68]. Although many efforts have been addressed to the biophysical characterization of many ALS-related SOD1 mutants, the molecular mechanisms by which the mutations cause fALS are still poorly understood. Investigations on the toxic function acquired by SOD1 have been focused on a prominent feature observed in both human patients and animal models of ALS: the accumulation of SOD1-rich proteinaceous aggregates containing SOD1 in the spinal cord. These observations have led to the hypothesis that SOD1 wild-type or mutants become unstable and misfold to form high-molecular-weight aggregates that are selectively toxic to motor neurons [69,70].

SOD1 aggregation is likely a consequence of the formation of misfolded SOD1 species in the spinal cords of fALS patients as well as presymptomatic mice expressing SOD1 mutants ALS-related [71,72]. Misfolded soluble SOD1 is selectively enriched in the spinal cords of these mice during their lifetime, suggesting that these species are sequestered in protein inclusions when neuronal cells are no longer able to handle the damage induced by misfolded SOD1 [73,74].

SOD1 mutants associated to fALS may be classified into two groups: the “wild-type like” and the “metal binding region” mutants [75]. In the “wild-type like” mutants, the mutations are scattered throughout the β-barrel of the protein and the zinc levels are very similar to those found in the wild-type protein, i.e., high in zinc but with variable levels of copper. Most of these mutants (i.e., E100K, D101N, D125H, S134N, N139K, D90A and N86S) are nearly indistinguishable from the wild-type protein in terms of stability, metal coordination properties and conformational kinetics as suggested by H/D exchange studies [35,76]. Nonetheless, where data are available, these variants have been shown to aggregate more readily than the wild-type protein in transgenic mouse lines, in cell cultures and in vitro [33,77]. A possible explanation for such behavior is that such mutations could induce minor conformational changes that lead to the formation of a native-like state more susceptible to self-association in vivo. In this type of process, the conformational changes that usually precede aggregation, such as local or global unfolding and other structural rearrangements, seem to occur after the formation of the native-like oligomers [78]. In this respect, the “wild-type like” mutations could favor the formation of native-like oligomers that promote protein aggregation. Moreover, other factors as the net surface charge could strongly contribute to protein aggregation propensity. Indeed, several ALS-related SOD1 mutations (i.e., D101N, E100K, N139K and D90A) induce a decrease of the net negative charge of the SOD1 polypeptide. In these mutants, aggregation is likely promoted by the reduction in net charge of the SOD1 polypeptide instead of destabilization of the native state. In addition, it is reasonable that stable SOD1 variants with a lower net charge might interact more readily with anionic membrane surfaces such as those present in the mitochondrial membrane thus inducing cytotoxicity [33].

In the “metal binding region” mutants, the mutations affect the metal binding ligands themselves or elements intimately associated with metal binding. When isolated from the expression systems, these mutants are characterized by very low zinc and copper content [27,29]. The occupation of copper and zinc binding sites not only affects the stability of SOD1 but it is also likely to act upon the free energy barrier to unfolding, thus affecting the so-called “kinetic stability” of SOD1 [32,36]. Therefore, the loss of these ions may facilitate partial or global unfolding transitions that may lead to protein misfolding and aggregation. In this respect, metal binding could play a key role in the aggregation process of SOD1 in fALS.

## 4. Metal Binding and Aggregation of SOD1 in ALS

SOD1 is a highly stable homodimeric protein that possesses in each monomer a disulfide bridge as well as a copper/zinc binuclear site. The remarkable stability and the metal-binding abilities of the wild-type SOD1 apoprotein do not seem to be required for the optimization of the superoxide dismutase activity [79]. Instead, it appears that these unique properties evolved to optimize the free-energy landscape of folding, disulfide bond formation, and efficient in vivo metalation of the immature disulfide-reduced nascent SOD1 apoprotein [36]. Particularly relevant in this regard are recent demonstrations of the strong influence of metal ions on the kinetics of SOD1 folding [41,80,81,82]. In addition, Leinartaite and coworkers have recently demonstrated that Zn^2+^ catalyzes the folding of apo-SOD1 by transient docking at the native copper site followed by a slower migration to the native zinc site after that latter site has formed [83]. Both metal binding and disulfide bond formation strongly contribute to the high stability of native SOD1 and these post-translational modifications are not independent but linked. Indeed, deficiency in metal binding increases the susceptibility of SOD1 to disulfide reduction [26]. In this respect, the native metalation and, in particular, the correct Zn^2+^ coordination plays a key role in the regulation of SOD1 folding [52,84].

Interestingly, SOD1 aggregates from transgenic fALS mouse models were found to be metal-deficient and/or lack the disulfide bond, raising the possibility that the in vivo aggregation of SOD1 could involve the immature forms of the protein lacking one or more post-translational modifications [34,72]. In addition, in vitro experiments revealed that the formation of monomeric SOD1 precedes the appearance of visible aggregates [85]. As for both SOD1 wild-type and most ALS-related mutants only the metal-free, disulfide-reduced form is monomeric [13,15,50]; therefore, many studies have been focused on in vitro aggregation of SOD1 lacking the metal cofactors and/or the intramolecular disulfide bond.

Comparison of the structure and mobility of apo-SOD1 and disulfide-oxidized holo-SOD1 suggests partial unfolding of apo-SOD1. Indeed, both the “zinc loop” and the “electrostatic loop” resulted significantly disordered in apo-SOD1 as suggested by X-ray crystallography and H/D exchange studies [32,86,87]. In addition, the disulfide-reduced apoprotein in vitro displays an increased surface hydrophobicity and a decreased mobility than the disulfide-oxidized holoprotein and recent molecular dynamics simulations support the hypothesis that local unfolding of the metal-free, disulfide-reduced SOD1 leads to the “building blocks” for SOD1 aggregation [26,88].

In vitro studies have shown that, while metalated SOD1 did not form amyloid-like aggregates at neutral pH, either removing metals from SOD1 with its intramolecular disulfide bond intact or reducing the intramolecular disulfide bond of metalated SOD1 was sufficient to promote formation of these aggregates [27,32,89]. This vulnerability of the apo-state to self-associate is related with high flexibility and disorder of both zinc and electrostatic loops [86,87]. Demetalated SOD1 formed amyloid-like aggregates in physiological conditions of pH, temperature and ionic strength regardless of the disulfide’s redox state suggesting that removal of copper and zinc was sufficient to trigger amyloid formation [25,27].

A significant fraction of wild-type SOD1 has been shown to be incompletely metalated in vivo [90,91,92], and both incompletely metalated and disulfide-reduced SOD1 are present in transgenic mice expressing various ALS mutants of SOD1 [73,77]. Intriguingly, several studies have shown that the presence of undermetalated and disulfide-reduced SOD1 is exacerbated by the presence of mutations related to ALS. Most of these mutations have been shown to promote amyloid formation in vitro by allowing dissociation of metals from SOD1 at pH values where metals remain tightly bound to wild-type SOD1, and this effect was most pronounced for the metal-binding-region mutants [25,91]. In this respect, the metal-binding-region mutations may promote amyloid aggregation in SOD1 by facilitating the loss of metals and/or by making the intramolecular disulfide bond more susceptible to reduction. In addition, several wild-type-like mutants, including the severe A4V and L38V, were shown to have lower zinc affinities than the wild-type protein in the presence of 2 M urea [92]. These observations suggest that, in a physiological context, loss of metals could be promoted by a local acidic environment or by other cellular stresses, and this would occur more readily with SOD1 mutants. Alternatively, newly synthesized mutant SOD1 proteins may form amyloid aggregates before metals are coordinated.

Although it is now widely accepted that low metalation could be a triggering factor in the ALS-related aggregation of SOD1, many studies have been recently focused on the role of aberrant metal binding in the aggregation process of SOD1. Indeed, SOD1 metal binding sites possess very different geometries, ligand types, labilities, and stabilities, and formation of non-native metal binding has been shown to occur in vitro [36,53,83,93]. In addition, mismetalation has been shown to trigger SOD1 toxic deposition [53]. For instance, zinc is known to have a promiscuous behavior towards SOD1 metal binding sites, being able to jump between the copper and zinc ligands through a parallel mechanism with undistinguishable energetics [83]. At the early stages of SOD1 folding, Zn^2+^ binds to the copper site accelerating the folding reaction. As the reaction progresses zinc binding site, which structures late in the folding and process, organized Zn^2+^ is transferred to its most thermodynamically favorable condition at the high affinity zinc site. If the zinc ligands are mutated, copper ligands can initially coordinate the non-native zinc ion; however, without the structural support of the loaded zinc site the protein misfolds and has an increased propensity to aggregate [83]. Thus, the aberrant zinc binding to immature conformers of metal-free SOD1 triggers the formation of protein aggregates and, considering that Zn^2+^ is upregulated in ALS affected motor neurons, the aberrant zinc-protein interactions may be one of the factors contributing to the onset of SOD1 pathological aggregation [53]. Likewise, Ni^2+^can bind to both metal binding sites of apo-SOD1 in vitro and this non-native binding has been shown to promote SOD1 misfolding and aggregation [36]. Also Ca^2+^ has been shown to bind SOD1 under physiological pH and induce conformational changes that promote aggregation in SOD1 [93]. As a ligand, Ca^2+^ is particularly versatile because it binds sites with irregular geometry, thus facilitating its nonspecific association to proteins [94]. In agreement, a bound Ca^2+^ ion was described in a crystal structure of a SOD1 clinical mutant although sequence analysis and bioinformatics failed to identify any canonical Ca^2+^ binding motif within SOD1 [95]. As Ca^2+^ is increased in ALS and is particularly abundant in the specific motor neurons affected in this disease, it may be considered between the factors contributing to the onset of SOD1 in vivo aggregation.

Taken together these data provide evidence that metal binding, in addition of being necessary for SOD1 enzymatic activity, is a key factor in the aggregation process of SOD1. In particular, both demetalation and aberrant metal binding have been shown to promote misfolding and aggregation in SOD1 suggesting a possible role of metal binding in SOD1 pathological aggregation. This modulation effect by a metal ion has been also demonstrated for other proteins involved in neurodegenerative disorders, highlighting the influence of the chemical neuronal environment with respect to metal ion homeostasis in pathologic aggregation processes [96].

## 5. Outlook

Protein misfolding and aggregation are common hallmarks in many neurodegenerative diseases. The propensity of a protein to aggregate is a complex property reflecting a combination of different biophysical and physico-chemical parameters such as net charge, hydrophobicity, native state stability and intrinsic β-structure as well as post translational modifications [97,98]. Copper-zinc superoxide dismutase, due to its complexity, is a suitable model for dissecting the role of physico-chemical properties on the aggregation propensity of a polypeptide chain. In particular, SOD1 is an extremely stable protein when fully assembled, but it is rather unstable and prone to aggregation in its metal-free form. As SOD1 is implicated in the formation of proteinaceous toxic aggregates in the affected motor neurons of familial and sporadic forms of ALS, metals are suggested to potentially modulate toxic protein deposition in ALS as well as in other neurodegenerative conditions. In addition, recent advances indicating that aberrant metal coordination is also able to promote SOD1 aggregation. This condition is likely to occur in vivo as perturbation on the intracellular concentrations of metal ions is known to be involved in neurodegenerative diseases, highlighting the influence of metal ion homeostasis in pathologic aggregation processes. Indeed, significantly increased level of zinc and copper ions as well as the other metal ions was reported in ventral areas of spinal cords from sporadic ALS cases suggesting a key role of metal dyshomeostasis in the pathogenesis of SOD1-ALS [99,100]. Interestingly, the treatment of mice expressing SOD pathological mutants with metal chelators was able to delay the disease onset and extend the mice lifespan [100,101,102]. The modulation effect by metal ions has been also demonstrated for other proteins involved in neurodegenerative disorders, highlighting the influence of the chemical neuronal environment, with regard to metal ion homeostasis, in pathologic aggregation processes. In this respect, normalizing metal dyshomeostasis in pathological conditions could be a key to developing therapeutics for these devastating diseases.
